# The Wide Complex Tachycardia Formula: Derivation and validation data

**DOI:** 10.1016/j.dib.2019.103924

**Published:** 2019-04-17

**Authors:** Adam M. May, Christopher V. DeSimone, Anthony H. Kashou, David O. Hodge, Grace Lin, Suraj Kapa, Samuel J. Asirvatham, Abhishek J. Deshmukh, Peter A. Noseworthy, Peter A. Brady

**Affiliations:** a Department of Cardiovascular Diseases, Mayo Clinic, USA; b Department of Medicine, Mayo Clinic, USA; c Department of Department of Health Sciences Research, Mayo Clinic, USA

## Abstract

A recent publication (May et al., 2019) introduced a novel means (i.e. WCT Formula) to automatically distinguish ventricular tachycardia and supraventricular wide complex tachycardia using modern-day computerized electrocardiogram software measurements. In this article, a summary of data components relating to the derivation and validation of the WCT Formula is presented.

**Table:** Specifications table

Subject area	Cardiology
More specific subject area	Electrocardiology
Type of data	Tables and figures of analyzed data
How data was acquired	Review of health records and automated measurements provided by computerized electrocardiogram interpretation software
Data format	Analyzed
Experimental factors	Paired wide complex tachycardia and subsequent baseline electrocardiograms were acquired within clinical settings at the Mayo Clinic Rochester or Mayo Clinic Health System of South Eastern Minnesota between September 2011 and November 2016.
Experimental features	In a two-part investigation, a logistic regression model (i.e. WCT Formula), comprised of computerized electrocardiogram measurements and novel computations, was derived and validated using two separate patient cohorts.
Data source location	Mayo Clinic, Rochester MN
Data accessibility	Featured data within this article.
Related research article	May, A. M., C. V. DeSimone, A. H. Kashou, D. O. Hodge, G. Lin, S. Kapa, S. J. Asirvatham, A. J. Deshmukh, P. A. Noseworthy, and P. A. Brady. 2019. ‘The WCT Formula: A novel algorithm designed to automatically differentiate wide-complex tachycardias', *J Electrocardiol*, 54: 61–68.

**Table:** 

**Value of the data** ○ Data would be valuable to researchers interested in specifying desired clinical and electrocardiogram (ECG) features to be evaluated in prospective studies which aim to accurately differentiate ventricular tachycardia (VT) and supraventricular wide complex tachycardia (SWCT). ○ Data would be valued by researchers interested in understanding patient demographics, clinical characteristics and electrocardiographic features of wide complex tachycardia (WCT) events encountered in clinical practice. ○ Enclosed data summarizes the patient demographics, clinical characteristics and ECG laboratory interpretation data of patient cohorts used to derive and validate the WCT Formula. ○ Enclosed data details the distribution of shared and non-shared WCT diagnoses between the WCT Formula, ECG laboratory interpretation and clinical diagnosis. ○ Enclosed data summarizes electrocardiographic characteristics of WCTs erroneously classified by the WCT Formula.

## 1 Data

Table 1 describes the clinical and ECG laboratory diagnosis data for the derivation cohort. Most (86.1%) clinical diagnoses were established by heart rhythm or non-heart rhythm cardiologists. A sizeable majority (91.8%) of WCTs were assigned definitive or probable interpretive diagnoses by the ECG laboratory. More than half of evaluated WCTs (51.4%) were derived from patients who underwent an electrophysiology procedure and/or possessed an implantable intra-cardiac device.

**Table 1:** Derivation cohort: Clinical and ECG laboratory diagnosis.^a^

	SWCT (n = 160)	VT (n = 157)	P value
Diagnosing Provider			
Heart rhythm cardiologists	70 (43.8)	147 (93.6)	<0.001
Non-Heart rhythm cardiologists	51 (31.9)	5 (3.2)	
Non-cardiologists	39 (23.4)	5 (3.2)	
Time Separation between WCT and Baseline ECG (hours)			
Mean (SD)	601.2 (2975.91)	176.7 (704.1)	0.54
Median	12.2	9.7	
Q1, Q3	1.4, 60.5	1.0, 53.4	
Range	0.0–29800.2	0.0–5307.5	
Time Separation between WCT and Baseline ECG			
<3 hours	58 (36.3)	64 (40.8)	0.41
3–24 hours	43 (26.8)	32 (20.4)	0.17
1–30 days	41 (25.6)	55 (35.0)	0.07
>= 30 days	18 (11.3)	6 (3.8)	0.01
ECG Lab Interpretation			
Definite VT	5 (3.1)	122 (77.7)	<0.001
Probable VT	13 (8.1)	20 (12.7)	
Definite SWCT	115 (71.9)	3 (1.9)	
Probable SWCT	10 (6.3)	3 (1.9)	
Undifferentiated	17 (10.6)	9 (5.7)	
Electrophysiology Procedure			
Yes	24 (15.0)	81 (51.6)	<0.001
Implantable Device			
Yes	20 (12.5)	109 (69.4)	<0.001

a Numbers in parentheses are percent (%) of n or standard deviation. SD = standard deviation; SWCT = supraventricular tachycardia; VT = ventricular tachycardia.

Table 2 summarizes the patient characteristics of the derivation cohort. The SWCT group included fewer ECG pairs from patients with coronary artery disease, prior myocardial infarction, prior cardiac surgery, ongoing antiarrhythmic drug use, ischemic cardiomyopathy, non-ischemic cardiomyopathy, and implanted cardioverter-defibrillator. Baseline ECGs with ventricular pacing were more common in the VT group. Preexisting bundle branch block was more prevalent in the SWCT group.

**Table 2:** Derivation cohort: Clinical characteristics .^a^

	SWCT (n = 160)	VT (n = 157)	P value
Age (years)			
Mean (SD)	71.5 (13.3)	66.1 (13.6)	0.002
Range	22–98	30–90	
Gender			
Male	99 (61.9)	127 (80.9)	<0.001
Female	61 (38.1)	30 (19.1)	
Clinical Characteristics			
Coronary artery disease	77 (48.1)	103 (65.6)	0.002
Prior myocardial infarction	44 (27.5)	88 (56.1)	<0.001
Prior cardiac surgery	52 (32.5)	71 (44.2)	0.02
Congenital heart disease	7 (4.4)	14 (8.9)	0.10
Anti-arrhythmic drug use	16 (10.0)	95 (60.5)	<0.001
Ischemic cardiomyopathy	29 (18.1)	74 (47.1)	<0.001
Non-ischemic cardiomyopathy	39 (24.4)	54 (34.4)	0.05
AICD	7 (4.4)	106 (67.5)	<0.001
Pacemaker	13 (8.1)	3 (1.9)	0.01
Left Ventricular Ejection Fraction (%)			
LVEF (>= 50)	90 (56.3)	33 (21.0)	<0.001
LVEF (49–31)	25 (15.6)	46 (29.3)	
LVEF (<= 30)	42 (26.3)	78 (49.7)	
Unknown LVEF	3 (1.9)	0 (0.0)	
Baseline ECG			
Baseline bundle branch block	102 (63.8)	27 (17.2)	<0.001
Baseline ventricular pacing	10 (6.3)	69 (44.0)	<0.001

a Numbers in parentheses are percent (%) of n or standard deviation. AICD = automatic implantable cardioverter-defibrillator; LVEF = left ventricular ejection fraction; SD = standard deviation; SWCT = supraventricular tachycardia; VT = ventricular tachycardia.

Table 3 describes the clinical and ECG laboratory diagnosis data for the validation cohort. Most (85.2%) clinical diagnoses were established by heart rhythm or non-heart rhythm cardiologists. Nearly all (98.2%) interpreted WCTs were assigned definitive or probable diagnoses by the ECG laboratory. A minority (31.0%) of evaluated WCTs were derived from patients who underwent an electrophysiology procedure. A sizable fraction (35.6%) of evaluated WCTs possessed an implantable intra-cardiac device.

**Table 3:** Validation cohort: Clinical and ECG laboratory diagnosis.^a^

	SWCT (n = 168)	VT (n = 116)	P value
Diagnosing Provider			
Heart rhythm cardiologists	71 (42.3)	101 (87.1)	<0.001
Non-Heart rhythm cardiologists	58 (34.5)	12 (10.3)	
Non-cardiologists	39 (23.2)	3 (2.6)	
Time Separation between WCT and Baseline ECG (hours)			
Mean (SD)	172.7 (900.8)	137.8 (522.2)	0.42
Median	5.0	5.4	
Q1, Q3	0.7, 28.2	1.0, 45.3	
Range	0.02–10097.1	0.1–4383.9	
Time Separation between WCT and Baseline ECG			
<3 hours	76 (45.2)	47 (40.5)	0.43
3–24 hours	45 (26.8)	31 (26.7)	0.99
1–30 days	37 (22.0)	32 (26.6)	0.28
>= 30 days	10 (6.0)	6 (5.2)	0.78
ECG Lab Interpretation			
Definite VT	5 (3.0)	104 (89.7)	<0.001
Probable VT	3 (1.8)	6 (5.2)	
Definite SWCT	150 (89.3)	3 (2.6)	
Probable SWCT	6 (3.6)	2 (1.7)	
Undifferentiated	4 (2.4)	1 (0.9)	
Electrophysiology Procedure			
Yes	27 (16.1)	61 (52.6)	<0.001
Implantable Device			
Yes	29 (17.3)	72 (62.1)	<0.001

a Numbers in parentheses are percent (%) of n or standard deviation. SD = standard deviation; SWCT = supraventricular tachycardia; VT = ventricular tachycardia.

Table 4 summarizes the patient characteristics of the validation cohort. The VT group included more ECG pairs from patients with coronary artery disease, prior myocardial infarction, ongoing antiarrhythmic drug use, ischemic cardiomyopathy, and implanted cardioverter-defibrillator. The SWCT included more ECG pairs from patients with an implanted pacemaker lacking cardioverter-defibrillator capability. Baseline ECGs with ventricular pacing were more common in the VT group. Preexisting bundle branch block was more prevalent in the SWCT group.

**Table 4:** Validation cohort: Clinical characteristics.^a^

	SWCT (n = 168)	VT (n = 116)	P value
Age (years)			
Mean (SD)	69.8 (15.8)	65.4 (12.4)	<0.001
Range	18–92	27–88	
Gender			
Male	113 (67.3)	98 (85.5)	0.001
Female	55 (32.7)	18 (15.5)	
Clinical Characteristics			
Coronary artery disease	83 (49.4)	85 (73.3)	<0.001
Prior myocardial infarction	49 (29.2)	69 (59.5)	<0.001
Prior cardiac surgery	71 (42.3)	47 (40.5)	0.77
Congenital heart disease	11 (6.6)	5 (4.3)	0.42
Anti-arrhythmic drug use	36 (21.4)	70 (60.3)	<0.001
Ischemic cardiomyopathy	23 (13.7)	64 (55.2)	<0.001
Non-ischemic cardiomyopathy	38 (22.6)	35 (30.2)	0.15
AICD	15 (8.9)	70 (60.3)	<0.001
Pacemaker	14 (8.3)	2 (1.7)	0.02
Left Ventricular Ejection Fraction (%)			
LVEF (>= 50)	99 (58.9)	36 (31.0)	<0.001
LVEF (49–31)	34 (20.2)	39 (33.6)	
LVEF (<= 30)	24 (14.3)	40 (34.5)	
LVEF Unknown	11 (6.6)	1 (0.9)	
Baseline ECG			
Baseline bundle branch block	115 (68.5)	12 (10.3)	<0.001
Baseline ventricular pacing	9 (5.4)	41 (35.3)	<0.001

a Numbers in parentheses are percent (%) of n or standard deviation. AICD = automatic implantable cardioverter-defibrillator; LVEF = left ventricular ejection fraction; SD = standard deviation; SWCT = supraventricular tachycardia; VT = ventricular tachycardia.

Table 5 provides a comparative analysis of clinical and ECG laboratory interpretation data for the derivation and validation cohorts. The validation cohort included more WCTs with definitive or probable interpretive diagnoses coded by the ECG laboratory (validation cohort: 98.2% vs. derivation cohort: 91.8%).

**Table 5:** Derivation vs. Validation Cohort: Clinical and ECG Laboratory Diagnosis.^a^

	Derivation Cohort (n = 317)	Validation Cohort (n = 284)	P value
Diagnosing Provider			
Heart rhythm cardiologists	217 (68.5)	172 (60.6)	0.08
Non-Heart rhythm cardiologists	56 (17.7)	70 (24.7)	
Non-cardiologists	44 (13.9)	42 (14.8)	
Time Separation between WCT and Baseline ECG (hours)			
Mean (SD)	391.0 (2178.5)	158.5 (768.1)	0.03
Median	10.7	5.2	
Q1, Q3	1.2, 53.4	0.8, 40.5	
Range	0.0–29800.2	0.02–10097.1	
Time Separation between WCT and Baseline ECG			
<3 hours	122 (38.5)	123 (43.3)	0.23
3–24 hours	75 (23.7)	76 (26.8)	0.38
1–30 days	96 (30.3)	69 (24.3)	0.10
>= 30 days	24 (7.6)	16 (5.6)	0.34
ECG Lab Interpretation			
Definite VT	127 (40.1)	109 (38.4)	<0.001
Probable VT	33 (10.4)	9 (3.2)	
Definite SWCT	118 (37.2)	153 (53.9)	
Probable SWCT	13 (4.1)	8 (2.8)	
Undifferentiated	26 (8.2)	5 (1.8)	
Electrophysiology Procedure			
Yes	105 (33.1)	88 (31.0)	0.58
Implantable Device			
Yes	129 (40.7)	101 (35.6)	0.20

a Numbers in parentheses are percent (%) of n or standard deviation. SD = standard deviation; SWCT = supraventricular tachycardia; VT = ventricular tachycardia.

Table 6 provides a comparative summary of the patient characteristics for the derivation and validation cohorts. The derivation cohort included more ECG pairs from patients with severely reduced LVEF (<30%). The derivation cohort included more ECG pairs with a ventricular paced baseline heart rhythm.

**Table 6:** Derivation vs. Validation Cohort: Patient Characteristics.^a^

	Derivation Cohort (n = 317)	Validation Cohort (n = 284)	P value
Age (years)			
Mean (SD)	68.8 (13.7)	68.0 (14.6)	0.93
Range	22–98	18–92	
Gender			
Male	226 (71.3)	211 (74.3)	0.41
Female	91 (28.7)	73 (25.7)	
Clinical Characteristics			
Coronary artery disease	180 (56.8)	168 (59.2)	0.56
Prior myocardial infarction	132 (41.6)	118 (41.6)	0.98
Prior cardiac surgery	123 (38.8)	118 (41.6)	0.49
Congenital heart disease	21 (6.6)	16 (5.6)	0.61
Anti-arrhythmic drug use	111 (35.0)	106 (37.3)	0.56
Ischemic cardiomyopathy	103 (32.5)	87 (30.6)	0.62
Non-ischemic cardiomyopathy	93 (29.3)	73 (25.7)	0.32
AICD	113 (35.7)	85 (29.9)	0.14
Pacemaker	16 (5.1)	16 (5.6)	0.75
Left Ventricular Ejection Fraction (%)			
LVEF (>= 50)	123 (38.8)	135 (47.5)	<0.001
LVEF (49–31)	71 (22.4)	73 (25.7)	
LVEF (<= 30)	120 (37.9)	64 (22.5)	
Unknown LVEF	3 (1.0)	12 (4.3)	
Baseline ECG			
Baseline bundle branch block	129 (40.7)	127 (44.7)	0.32
Baseline ventricular pacing	79 (24.9)	50 (17.6)	0.03

a Numbers in parentheses are percent (%) of n or standard deviation. AICD = automatic implantable cardioverter-defibrillator; LVEF = left ventricular ejection fraction; SD = standard deviation; SWCT = supraventricular tachycardia; VT = ventricular tachycardia.

Table 7 summarizes the electrocardiographic characteristics of SWCTs erroneously classified as VT by the WCT Formula's 50% VT probability partition.

**Table 7:** Electrocardiographic characteristics of clinical VTs classified as SWCT by the WCT Formula's 50% VT probability partition.^a^

WCT Formula Diagnosis	Clinical Diagnosis	ECG Laboratory Diagnosis	WCT Formula VT Probability (%)	Frontal PAC (%)	Horizontal PAC (%)	Baseline ECG QRS Duration	Baseline ECG Frontal QRS axis (°)	Baseline ECG V1 QRS Morphology	Baseline ECG V6 QRS Morphology	Baseline ECG Precordial Transition	WCT QRS Duration (ms)	WCT Frontal QRS axis (°)	WCT V1 QRS Morphology	WCT V6 QRS Morphology	WCT Precordial Transition
SWCT	VT	Probable SWCT	48.438	46.650	111.071	116	−8	rS	qR	V6	140	57	rS	RS	V6
SWCT	VT	Definite VT	47.651	28.424	53.163	136	−15	RSR	RS	V2	184	−32	R	QRs	None
SWCT	VT	Definite VT	47.242	24.642	80.935	188	52	rS	R	V4	168	72	rS	qR	V4
SWCT	VT	Definite VT	44.548	44.399	108.675	170	−86	R	rS	V3	140	−63	R	RS	None
SWCT	VT	Definite VT	31.683	99.149	81.050	98	−18	rS	qR	V4	124	−51	QS	Rs	V2
SWCT	VT	Definite VT	26.868	35.826	85.223	88	−38	rS	qRs	V2	146	−36	rS	QS	V4
SWCT	VT	Definite VT	23.134	65.972	75.964	118	74	rS	qR	V5	136	29	QS	R	V3
SWCT	VT	Definite VT	18.814	37.407	78.684	84	−61	rS	RS	V4	142	−81	R	RS	V5
SWCT	VT	Definite VT	18.503	114.122	48.912	132	18	rS	qRs	V3	126	−45	rSr	qrS	V2
SWCT	VT	Definite VT	6.290	65.530	55.606	140	84	rSr	Rs	V4	124	81	QS	R	V2
SWCT	VT	Definite VT	3.560	81.152	19.608	110	−51	rSr	RS	V6	130	−28	QS	RS	V5
SWCT	VT	Definite VT	3.125	29.765	49.641	110	−59	rS	qRs	V3	132	−61	QS	RS	V5

a ECG = electrocardiogram; PAC = percent amplitude change; SWCT = supraventricular wide complex tachycardia; VT = ventricular tachycardia; WCT = wide complex tachycardia.

Table 8 summarizes the electrocardiographic characteristics of VTs erroneously classified as SWCT by the WCT Formula's 50% VT probability partition.

**Table 8:** Electrocardiographic characteristics of clinical SWCTs classified as VT by the WCT Formula's 50% VT probability partition.^a^

WCT Formula Diagnosis	Clinical Diagnosis	ECG Laboratory Diagnosis	WCT Formula VT Probability (%)	Frontal PAC (%)	Horizontal PAC (%)	Baseline ECG QRS Duration	Baseline ECG Frontal QRS axis (°)	Baseline ECG V1 QRS Morphology	Baseline ECG V6 QRS Morphology	Baseline ECG Precordial Transition	WCT QRS Duration (ms)	WCT Frontal QRSaxis (°)	WCT V1 QRS Morphology	WCT V6 QRS Morphology	WCT Precordial Transition
VT	SWCT	Definite VT	99.940	146.000	221.877	104	129	rS	qrs	V5	146	−76	rS	qR	V6
VT	SWCT	Definite SWCT	99.405	144.474	157.844	158	−82	QS	QS	None	150	118	rsR	RS	None
VT	SWCT	Definite VT	89.679	97.656	67.833	86	22	rS	Rs	V3	180	−62	rS	qRs	V5
VT	SWCT	Definite SWCT	88.953	84.193	142.417	136	−43	QS	qRs	V5	138	70	rS	R	V5
VT	SWCT	Definite SWCT	81.050	118.900	104.656	174	63	QS	R	V5	136	−28	qR	RS	None
VT	SWCT	Definite SWCT	78.889	84.320	62.144	122	−8	rS	qR	V5	176	−52	rS	Rsr	V6
VT	SWCT	Definite SWCT	77.680	84.173	85.923	118	1	rS	qR	V4	160	−35	rS	RS	V6
VT	SWCT	Definite SWCT	67.787	96.304	96.368	100	10	rS	Rs	V3	140	56	R	R	None
VT	SWCT	Definite SWCT	61.864	29.959	105.085	168	−28	QS	R	V6	160	−70	rS	rS	None
VT	SWCT	Definite SWCT	60.852	23.008	80.273	118	−46	rS	RS	V5	178	−52	rS	rS	None
VT	SWCT	Definite SWCT	56.498	97.282	41.799	108	19	rS	qR	V5	166	−37	rS	rSr	None
VT	SWCT	Definite SWCT	51.740	62.594	48.899	154	−19	QS	R	V5	174	−58	QS	QrS	None

a ECG = electrocardiogram; PAC = percent amplitude change; SWCT = supraventricular wide complex tachycardia; VT = ventricular tachycardia; WCT = wide complex tachycardia.

**Fig. 1:**
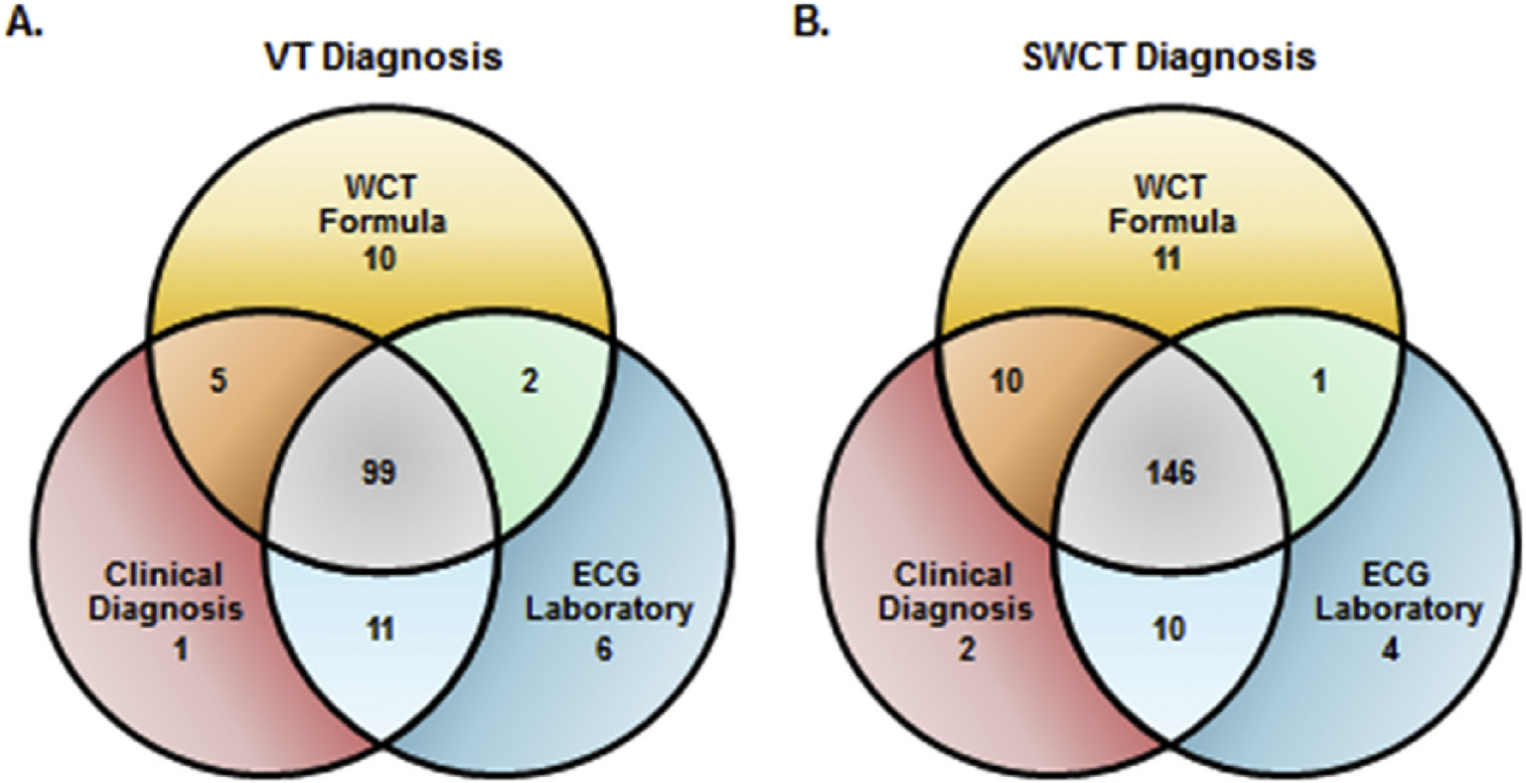
**Diagnostic Agreement**. Venn diagrams summarizing the distribution of VT (A) and SWCT (B) diagnoses established by (1) WCT Formula's 50% VT probability partition, (2) clinical diagnosis and (3) ECG laboratory interpretation. Undifferentiated WCT diagnoses (n = 5) established by the ECG laboratory are not shown.

Fig. 1 summarizes the distribution of shared and non-shared WCT diagnoses between (1) the WCT Formula's 50% VT probability partition, (2) clinical diagnosis and (3) ECG laboratory interpretation. The WCT Formula's agreement with VT diagnoses established by either or both the ECG laboratory and clinical diagnosis was 91.4% and 85.3%, respectively. The WCT Formula's agreement with SWCT diagnoses established by either or both the ECG laboratory interpretation and clinical diagnosis was 93.5% and 86.9%, respectively.

## 2 Experimental design, materials, and methods

A recent study by May and colleagues details the development and validation of a logistic regression model capable of automatic VT probability estimation [1]. In a two-part investigation, a logistic regression model (i.e. WCT Formula) was derived and validated using two separate patient cohorts. In Part 1, a derivation cohort of paired WCT and subsequent baseline ECGs was examined to identify independent VT predictors to be incorporated into the WCT Formula. In Part 2, the WCT Formula's performance was prospectively evaluated against a validation cohort of paired WCT and subsequent baseline ECGs. The derivation cohort was comprised of 317 paired WCT (157 VT, 160 SWCT) and baseline ECGs. The validation cohort consisted of 284 paired WCT (116 VT, 168 SWCT) and baseline ECGs. The diagnostic performance of the WCT Formula was appraised according to its agreement with clinical and/or ECG laboratory diagnosis.

**Fig. 2:**
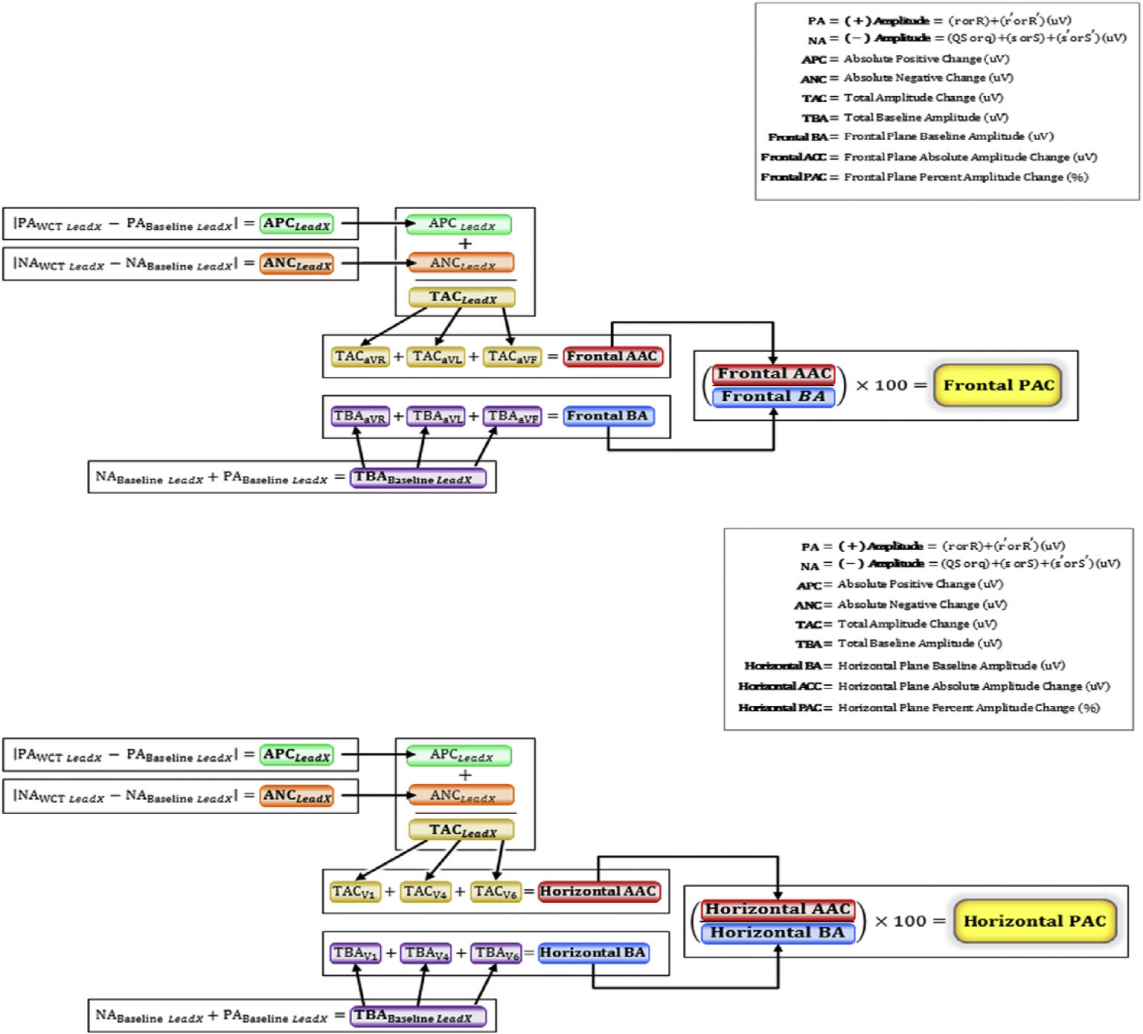
**Frontal and Horizontal PAC Calculations,** The frontal and horizontal PAC calculations are composed of measured QRS waveform amplitudes (μV) derived from select ECG leads within the frontal or horizontal plane. *LeadX* denotes individual ECG leads within the frontal (aVR, aVL, aVF) or horizontal (V1, V4, V6) ECG plane. Positive Amplitude (PA) is the sum of measured QRS waveform amplitudes above the isoelectric baseline (*r/R* and *r’/R′*) in a single ECG lead. Negative Amplitude (NA) is the sum of measured QRS waveform amplitudes below the isoelectric baseline (*q/QS*, *s/S*, and *s’/S′*) in a single ECG lead. Total Baseline Amplitude (TBA) is the sum of PA and NA within individual ECG leads of the baseline ECG. Baseline Amplitude (BA) is the summation of TBAs from select ECG leads in the frontal (aVR, aVL, aVF) or horizontal (V1, V4, V6) ECG planes. Absolute Positive Change (APC) and Absolute Negative Change (ANC) are an individual ECG lead's absolute QRS amplitude change above and below the isoelectric baseline, respectively. Total Amplitude Change (TAC) is the sum of APC and ANC within an individual ECG lead. Absolute Amplitude Change (AAC) is the combined sum of TACs from select ECG leads of the frontal (aVR, aVL, aVF) or horizontal (V1, V4, V6) ECG planes. Percent Amplitude Change (PAC) is the percent ratio of AAC to BA.

Paired WCT and subsequent baseline ECGs were acquired within clinical settings at the Mayo Clinic Rochester or Mayo Clinic Health System of South Eastern Minnesota between September 2011 and November 2016. Evaluated ECGs were standard, 12-lead recordings (paper speed: 25 mm/s, voltage calibration: 10 mm/mV) acquired from our institution's centralized ECG data archives (*GE Healthcare;* Milwaukee, WI). Data relating to clinical diagnosis, ECG laboratory interpretation and patient characteristics were recorded from the electronic medical record. Automated ECG measurements were accessed from *GE Healthcare's* MUSE ECG interpretation software. Novel computations, including frontal and horizontal percent amplitude change (PAC) (Fig. 2), were calculated using automated measurements derived from paired WCT and subsequent baseline ECGs.
